# Optimization of recurrent rapid cycle breeding in maize for sustained long-term genetic improvement via stochastic simulations

**DOI:** 10.1093/g3journal/jkaf100

**Published:** 2025-05-20

**Authors:** Torsten Pook, Mila Leonie Tost, Henner Simianer

**Affiliations:** Department of Animal Sciences, Animal Breeding and Genetics Group, University of Goettingen, 37075 Goettingen, Germany; Center for Integrated Breeding Research, University of Goettingen, 37075 Goettingen, Germany; Animal Sciences, Animal Breeding and Genomics Group, Wageningen University & Research, 6700 AH Wageningen, The Netherlands; Department of Animal Sciences, Animal Breeding and Genetics Group, University of Goettingen, 37075 Goettingen, Germany; Center for Integrated Breeding Research, University of Goettingen, 37075 Goettingen, Germany; Department of Crop Sciences, Plant Breeding and Methodology Group, University of Goettingen, 37075 Goettingen, Germany; Department of Animal Sciences, Animal Breeding and Genetics Group, University of Goettingen, 37075 Goettingen, Germany; Center for Integrated Breeding Research, University of Goettingen, 37075 Goettingen, Germany

**Keywords:** rapid cycle, breeding, simulation, maize, speed breeding, rapid generation advance, plantae

## Abstract

In recent years, the turnover of germplasm in plant breeding has substantially increased as the use of genomic information allows for earlier selection and the integration of controlled growing environments reduces the time to reach a particular growing stage. However, high generation turnover and intensive selection of lines before own yield trials are performed come at the risk of a drastic reduction of genetic diversity and lower prediction accuracies. To this end, we investigate strategies to cope with these challenges in a maize rapid cycle breeding scheme using stochastic simulations employing the software MoBPS. We find that genetic gains soon reach a plateau when only the original breeding material is phenotyped. Updating the training data set via additional phenotyping of crosses or doubled haploid lines ensures long-term progress with a gain of 6.80/6.95 genetic standard deviations (gSD) for the performance as a cross/DH after 30 cycles of breeding compared with 3.40/4.28 without additional phenotyping. Introducing genetic material from outside the breeding pool to introduce novel genetic diversity led to a further increase to 9.34/7.89 gSD. In particular, for the management of genetic diversity, further modifications of breeding program design are analysed to optimize the number of selected lines per cycle and to account for the relatedness of F2 plants in the selection using the software AlphaMate. Balancing short-term genetic gains with long-term diversity preservation is crucial for sustainable breeding. MoBPS provides a tool for quantifying these effects and provides solutions specific to the respective breeding program.

## Introduction

Plant breeding is a complex task with the ultimate goal of producing crop varieties with unique and superior traits, used in human and animal nutrition or as a renewable energy source. Breeders are continuously working on improving the characteristics of varieties to achieve sustainable and long-term genetic gain. With rising demands for higher production levels and more complex breeding goals, plant breeders face ongoing pressure to improve breeding strategies (Foley *et al.* 2011).

Genetic gain can be increased by controlled mating, which leads to an increase in heritability, higher selection intensity, or reduced time between generations (Falconer *et al.* 1996). The options for further increasing the selection intensity are limited, as this might reduce genetic diversity (Falconer *et al.* 1996). As a result, greater emphasis is placed on reducing the generation interval to enable multiple breeding cycles within a given time frame. To achieve this, large-scale breeding companies often ship breeding material between the northern and southern hemispheres to achieve 2 breeding cycles per calendar year. By the use of the controlled environment of a greenhouse with supplemental lighting, heating, and advanced modern crop breeding technologies the number of cycles per year can be increased even further, with speed-breeding approaches enabling up to 6 cycles for wheat, barley, and chickpea, or 4 cycles per year in canola (Ghosh *et al.* 2018; Watson *et al.* 2018). With the introduction of genomic selection (Meuwissen *et al.* 2001; Schaeffer 2006; Jannink 2010; Heslot *et al.* 2015), this process can be made even more efficient as reliable estimates of breeding value are available earlier and lines can be selected before own phenotypes are available, with approaches such as rapid cycle breeding (Gerald *et al.* 2013; Crossa *et al.* 2017; Gorjanc *et al.* 2018). Thus, rapid cycling paired with speed breeding has the potential to be a highly efficient framework for various applications in plant breeding like variety development, pre-breeding, and bridging efforts to introduce new genetic material into an existing breeding program (Gorjanc *et al.* 2016; Allier *et al.* 2020). In recent years, pre-breeding has become increasingly relevant for the utilization of genetic resources and for broadening the genetic basis of elite germplasm (Smith *et al.* 2015; Gaikpa *et al.* 2021). In this sense, pre-breeding can be a tool to make material of high genetic diversity useable for breeding (Mayer *et al.* 2018, 2020). However, as these concepts have only recently been enabled for applied use, practical yield trials and studies have only considered the use of these breeding schemes for a limited number of breeding cycles (Dreisigacker *et al.* 2023; Polzer *et al.* 2025). However, as modern varieties are much superior for performance traits like yield compared with more diverse landrace material or old breeding populations (Voss-Fels *et al.* 2019), it will be essential to pursue a rapid cycling breeding scheme for many generations to bridge the gap between exotic and elite breeding material (Breider *et al.* 2022).

This, in turn, comes with various challenges (Wanga *et al.* 2021)—particularly in the context of phenotyping. Phenotyping of some traits is only possible in adult plants and/or requires seed propagation steps (Watson *et al.* 2018). Thus, phenotypic data are less reliable or only available for plants that are already several cycles behind the current breeding material. This raises new questions such as whether and when genomic prediction models will break down as they are only supplied with phenotypic data from past breeding cycles, hence leading to lower prediction accuracies (Lorenz 2013; Neyhart *et al.* 2017), with (Polzer *et al.* 2025) reporting only limited genetic gain in the 3rd cycle of a rapid cycle breeding scheme in maize across multiple replicates. Additionally, genetic diversity might decrease with a high number of breeding cycles and further reduced prediction accuracy (Sallam *et al.* 2015; Schopp *et al.* 2017). Therefore, a detailed consideration concerning the management of genetic diversity and inbreeding is needed (Smith *et al.* 2015; Louwaars 2018).

Although quantitative genetic theory provides indications of the expected effect of breeding actions, changing one parameter in a breeding program inevitably affects other parameters. Hence, modern breeding programs are usually too complex to reliably assess the effect of a single isolated change in breeding program design (Pook *et al.* 2021b), in particular, if the relative effects are small compared with the underlying stochasticity of the breeding scheme. Therefore, breeding decisions to some extent rely on the breeder’s experience (Duvick 2002) that is often based on previous years and a limited number of real-world breeding experiments. As financial resources are limited, computer simulations (Sargolzaei and Schenkel 2009; Pook *et al.* 2020b; Gaynor *et al.* 2021; Villiers *et al.* 2022) can be a useful tool to aid the decision-making process in a breeding program and complement real-world breeding experiments.

Due to the increase in computational power in recent years, the use of stochastic simulations for quantitatively evaluating breeding programs using tools such as MoBPS (Pook *et al.* 2020b) has been suggested with various examples from practical breeding programs in animal breeding (Büttgen *et al.* 2020; Pook *et al.* 2021b; Hassanpour *et al.* 2023; Martin *et al.* 2023). Although the MoBPS framework has all the required features for the realistic modelling of plant breeding programs, there are, to date, no published exemplary applications of the framework for complex plant breeding programs.

An important component of long-term breeding is the management of genetic diversity with approaches like optimum contribution selection (Meuwissen 1997; Woolliams *et al.* 2015), which is being widely used, particularly in animal breeding. However, direct application in plant breeding is difficult, as it is for example common practice to work with a limited number of selected plants with equal contributions. To handle these kinds of restrictions, approaches such as AlphaMate (Gorjanc and Hickey 2018) and MateSel (Kinghorn 2011) have been proposed which suggest mating plans under a set of predefined constraints and optimize these via evolutionary algorithms. Thereby, providing a solution to optimum contribution selection that allows to incorporate more complex constraints than the direct solution derived via Lagrange-based optimization (Meuwissen 1997). Beyond this, more attention has been given to not only considering the expected performance of an offspring but also its variance, with approaches that consider Mendelian sampling variance (Bonk *et al.* 2016; Allier *et al.* 2019; Bijma *et al.* 2020; Niehoff *et al.* 2024)

In this work, we evaluate the potential of speed breeding exemplified by a rapid cycle breeding scheme in maize (*Zea mays*) by employing stochastic simulations in MoBPS (Pook *et al.* 2020b). A particular focus of the analysis is on the management of genetic diversity through the selection and introduction of new material with comparable genetic level from outside of the breeding pool. Furthermore, differences between direct selection on traits phenotyped in crosses compared with indirect selection by phenotyping of DH lines are analysed.

## Materials and methods

As a baseline scenario, we considered a breeding scheme inspired by the rapid cycle breeding scheme from the MAZE project (http://www.europeanmaize.net/) as first suggested by Polzer *et al.* (2025), aiming to use pre-breeding to enhance performance from material of a European maize landrace. In the project, which is not directly transferable to a commercial breeding program, only 3 plant height (PH) traits at different growth stages (V4, V6, final) were considered for selection; for details on the different growing stages, see Hölker *et al.* (2019). The heritabilities and genetic correlations between the traits were taken from Hölker *et al.* (2019). In MoBPS, the 3 traits were simulated in 2 variants, one representing the performance as an inbred line *per se* and the other performance as a test cross, hereafter referred to as DH and cross traits. Genetic correlations were taken from Hölker *et al.* (2019) as given in Table 1 with genetic correlations between DH and cross traits of different traits estimated based on available correlations. Environmental correlations were assumed to be small with used values given in Table 1. Whenever phenotyping was performed, the corresponding version (DH/cross) of the trait was phenotyped. Each trait was simulated to be polygenic with 500 purely additive QTLs, 500 purely dominant QTLs, 100 qualitative epistatic QTLs, and 100 quantitative epistatic QTLs each (n.additive, n.dominant, n.qualitative, n.quantitative in MoBPS Pook *et al.* 2020b). Due to the genetic correlation between traits, QTLs of each trait also impact the other traits. The interested reader is referred to MoBPS User Guidelines at https://github.com/tpook92/MoBPS and for details on the simulation of correlated traits in MoBPS.

**Table 1.** Heritabilites (main diagonal), genetic correlations (above diagonal), and environmental correlation (below diagonal) of the simulated traits.

	DH			cross		
Trait	PH_v4	PH_v6	PH_final	PH_v4	PH_v6	PH_final
PH_v4 (DH)	0.95	0.94	0.50	0.62	0.61*	0.25*
PH_v6 (DH)	0.30	0.95	0.58	0.56*	0.68	0.34*
PH_final (DH)	0.10	0.15	0.96	0.20*	0.30*	0.78
PH_v4 (cross)	0.20	0.06	0.02	0.76	0.86	0.21
PH_v6 (cross)	0.06	0.20	0.03	0.30	0.77	0.33
PH_final (cross)	0.02	0.03	0.20	0.10	0.15	0.87

Genetic correlations that were estimated based on available correlations are indicated with * using $\rho_{i,j}^{\mathrm{DH},\;\mathrm{cross}}=\sqrt{\rho_{i,j}^{\mathrm{DH}}\cdot \rho_{i,j}^{\mathrm{cross}}}\cdot \rho_{i,i}^{\mathrm{DH},\;\mathrm{cross}}$.

**Fig. 1.**
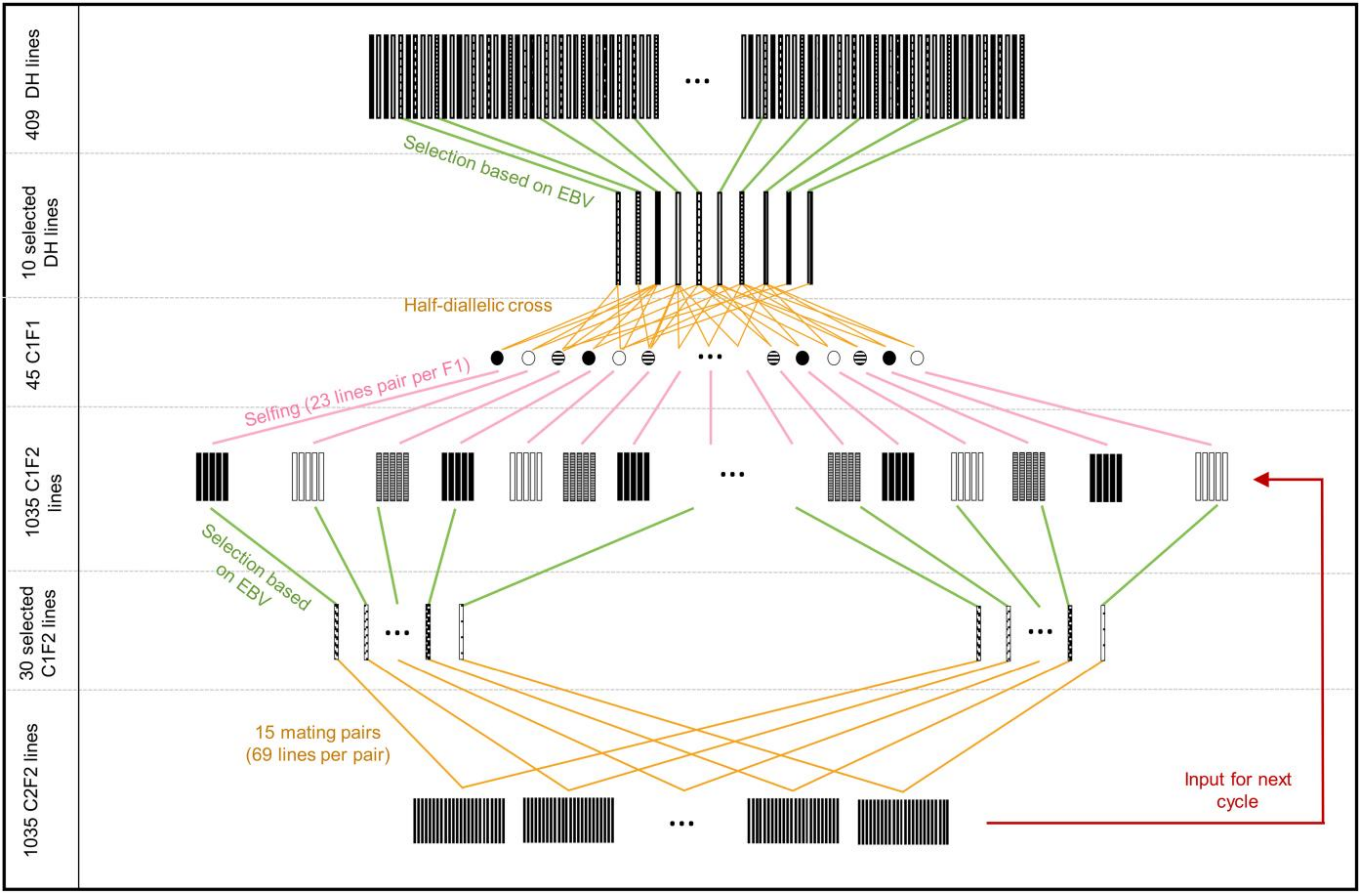
Schematic overview of a rapid cycle breeding scheme.

A schematic overview of the breeding program is given in Fig. 1. The breeding program as implemented in the MoBPS interactive environment from Pook *et al.* (2021a) is given in Supplementary Fig. S1. As a starting point of the breeding program, an initial pool of 409 founder DH lines of a European maize landrace was used (Mayer *et al.* 2020). Initially, 10 DHs were selected based on performance in the selection index with breeding values estimated via genomic best linear unbiased prediction (gBLUP) (Meuwissen *et al.* 2001) with phenotypes being simulated for the initial DHs. Subsequently, selected DHs were combined in a half-diallelic crossing scheme to generate offspring from all 45 possible genotype combinations. Crosses were subsequently selfed to generate 23 offspring from each combination, resulting in a pool of 1,035 F2 lines in cycle 1 (C1F2). From these, the best performing 30 C1F2 were selected based on a breeding value estimation based on the phenotypes from original DHs lines. From the top 30 lines, 15 crossing pairs were created at random and 69 offspring C2F2 lines were generated from each mating pair, resulting again in a pool of 1,035 lines. To mimic the generation of a final product of the breeding scheme, DH lines were produced from the top 3 F2 lines. To avoid a severe loss in genetic diversity, at most 10 C1F2 individuals that originated from the same initial DH line were selected in the first cycle. The R package rrBLUP was used for the estimation of breeding values (Endelman 2011).

In contrast to the real-world breeding program in the MAZE project (Polzer *et al.* 2025), where only 3 breeding cycles were considered, a considerably longer horizon of 30 breeding cycles was considered in this study, aiming for sustained long-term genetic improvement. Additionally, the selection of lines was done based on a selection index with equal weight on 3 traits, aiming to increase the first 2 traits and to decrease PH_final. In contrast, the real-world breeding scheme used the absolute difference from the current average plant height for PH_final with twice the weight instead of direct selection for low final PH (Polzer *et al.* 2025). This was done to avoid the effects of a long-term overall change in PH_final affecting the design of the index. In the following, we report only the results concerning the overall selection index.

In addition to the baseline scenario, various modifications of the breeding system were considered and are described in the following subsections. For all subsequently described scenarios, visualizations based on MoBPSweb (Pook *et al.* 2021a) can be found in Supplementary Figs. S2–S4.

### Additional phenotyping

Firstly, additional phenotyping of crosses (Supplementary Fig. S2) was considered. For simplicity, we did not simulate a second gene pool to mimic the mating to a tester and phenotyping as a hybrid. Instead, we simulated phenotypes based on the F2 genotypes directly with the appropriate heritability for crosses taken from Hölker *et al.* (2019). Since crosses are phenotyped in a plot and the same tester plant is commonly used for all lines this should not affect the results here. Since this requires an additional seed propagation step in practice, it was assumed that phenotypes for the selected F2 lines are available from 2 cycles ago. As a second strategy, the generation of DH lines from the F2 lines was considered with subsequent phenotyping of DHs for their *per se* performance (Supplementary Fig. S3). Given the cost of DH production and phenotyping, along with the expected technical failure rate (Chaikam *et al.* 2012; Melchinger *et al.* 2016), phenotypes were assumed to only be available for 200 of the 1,035 F2 lines. Since DH production in practice requires steps of induction, selfing, multiplication, and phenotyping it was assumed that phenotypes are only available for the lines from 4 cycles ago. The training population for the breeding value estimation included the last 4 generations of DHs with available phenotypes, resulting in a training population of 800 lines with phenotypes from DHs compared with the 1,035 phenotypes from crosses.

### Genetic diversity

For the management of genetic diversity, 3 strategies were considered: Firstly, the introduction of new genetic material from outside the breeding pool was considered. For this, new genetic material was simulated in each cycle by using the founder DH lines as a starting point and applying a mild selection intensity in a recurrent selection scheme (200 selected lines from a pool of 409 lines; Supplementary Fig. S4). Recurrent selection was performed until the average genetic value of the population exceeded the average genetic value in the F2 lines, with the index weights being adapted in each cycle to obtain similar genetic progress in each respective trait. Subsequently, material from the previous generation with slightly lower performance was used to replace the selected F2 lines in some of the crosses to generate the F2s of the next cycle. As a baseline, 20% of all matings used external material with various other proportions being considered (10%, 30%, 50%, 70%, 100%). For the breeding value estimation, 200 DH lines from the newly introduced material were generated and phenotyped. To maintain a constant training population size, the last 2 cycles of DH lines with available phenotypes from within the breeding program and DH lines from the last 2 newly introduced cohorts were considered, resulting in a training population of 800 DH lines with phenotypes. For scenarios where selection is based on phenotypes from crosses, no additional phenotypes from the newly introduced cohorts were generated.

Secondly, the use of optimum contribution selection (Woolliams *et al.* 2015), implemented via the software AlphaMate (Gorjanc and Hickey 2018) was used to identify the 15 required mating pairs. For this, each F2 line was limited to be used at most once (LimitContributionsMax=1) and the angle parameter in AlphaMate was used to balance between genetic gain and inbreeding. A low angle in AlphaMate puts a high weight on genetic gain, while a high angle will put more focus on preserving genetic diversity (TargetDegrees=10,20,30,40,50,60,70).

Lastly, the applied selection pressure was varied. For this, the number of selected lines was varied at 2 stages in the breeding scheme. Firstly, the number of originally selected DH lines from the initial founder set of 409 lines as the source of initial genetic diversity of the population was varied from 5 to 50 (baseline: 10). Secondly, the number of selected F2s per cycle from the pool of 1,035 lines was varied between 10 and 200 (baseline: 30).

### Evaluation metrics

Various metrics were used for the evaluation of simulation outcomes. The resulting genetic gain for the DH traits represents the genetic gain compared with the initial DH populations. The genetic gain as a cross is derived by comparison of F2 lines to crosses that were generated from the initial unselected DH lines. For the evaluation of the remaining genetic diversity, the variance of true underlying genomic values within the individuals of a cohort is calculated. Additionally, the share of heterozygous variants and the share of fixed markers in the F2s were computed. To evaluate the performance of the breeding value estimation, the correlation between the underlying true genomic values and the estimated breeding value, hereafter referred to as the prediction accuracy, is used.

All reported results are averages of 250 simulations per scenario.

## Results

**Fig. 2.**
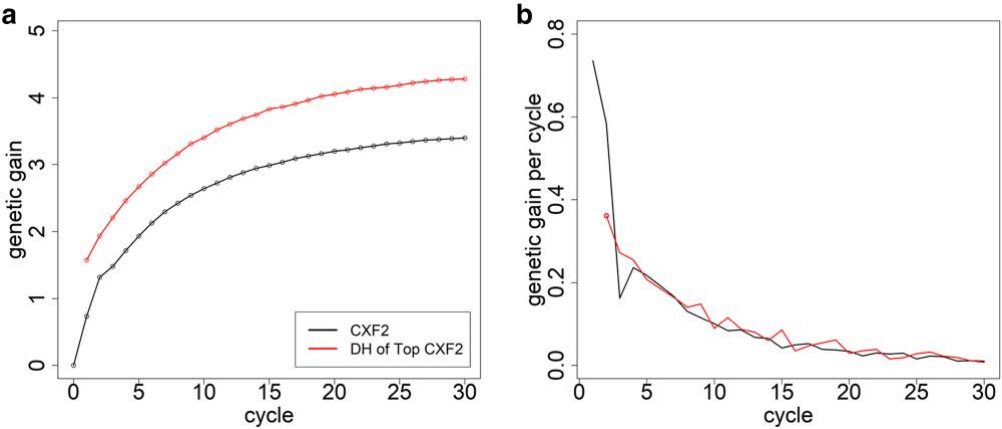
Genetic gain (in units of gSD) across the breeding cycles a) and per cycle b) within the F2 lines (CXF2) and DHs from the best F2 (CXF2-DH). The comparison of performance is against DH lines from the initial founder population and randomly generated crosses from the founders, respectively.

In the baseline rapid cycle scheme, the average genomic value of the F2 lines after 30 breeding cycles is increased by 3.40 genetic standard deviations (gSD). The average genomic value of the top DH lines is increased by 4.28 gSD compared with the initial DH lines (Fig. 2a).

**Fig. 3.**
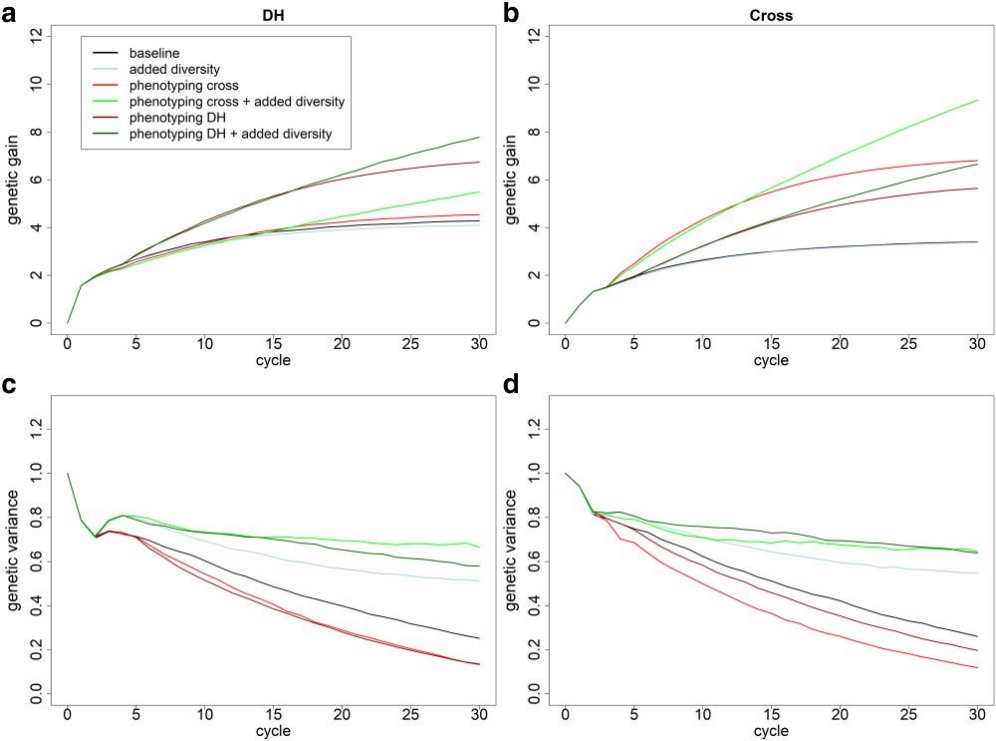
Genetic gain (in units of gSD) on the selection index for the crosses a) and DHs b), as well as the remaining genetic diversity (as variance of underlying true genomic values) of the crosses c) and DHs d) in the F2 lines of the respective cycle based on the chosen phenotyping strategy and action against inbreeding.

By far, the highest gains are obtained in the first 2 cycles. Genetic gains for traits expressed as a cross in cycle 3 are even slightly smaller than in subsequent cycles. After cycle 4, gains are slowly decreasing from around 0.25 gSD per cycle to 0.02 gSD per cycle in cycle 30 (Fig. 2b). Firstly, this is caused by a severe loss in genetic diversity as the gSD after 30 cycles is reduced by 74% compared with the initial population (Fig. 3c). Secondly, the genetic distance from the training population with available phenotypes is increasing with a reduced average prediction accuracy of 0.18 in cycle 30 compared with 0.72 in cycle 1 (Supplementary Fig. S5). Especially for the performance in crosses, a plateau is reached after a couple of cycles with minimal additional genetic gain with indirect selection on DH phenotypes (Fig. 2). The share of heterozygous genotypes reduces from 10.9% to 1.6%, with 94.3% of all markers being fixed after 30 generations (Supplementary Tables S1 and S2).

### Additional phenotyping

When additional phenotyping of the F2 lines was performed, a strong increase in the performance as a cross (6.80 gSD; + 100%) and a small improvement as a DH line (4.54 gSD; + 6%) was observed. The obtained prediction accuracies for the different cycles are about 0.55 (Supplementary Fig. S6). Although gains were smaller for the DH line performance, the indirect selection based on the phenotypes in crosses was still more efficient than the use of the phenotypes of the founder DHs. When instead generating phenotypes for additional DH lines, the average genetic gains were 5.69 gSD (+67%) and 6.74 (+57%) for DH lines from top F2 lines with average prediction accuracies of 0.60 across the different cycles (Supplementary Fig. S6).

It is to note that additional phenotyping led to an even stronger drop in the remaining genetic diversity. The standard deviations of the genetic values among the F2 lines is reduced by about 87% after 30 cycles (Fig. 3c).

### Genetic diversity through introduction of new material

When adding additional genetic material with similar genetic values to the current F2 population, this did not result in any additional genetic progress compared with the baseline scenario (Fig. 3) with genetic gains of 3.37/4.10 gSD (−1%/−4%). Genetic gains for both scenarios with additional phenotyping in the first few cycles were slightly worse because the newly introduced genetic material was genetically slightly inferior. However, after around 15–20 breeding cycles, genetic gains were on par with the scenario without newly introduced genetic material, with substantially higher gains in subsequent cycles. After 30 cycles genetic gains were 9.34/5.48 gSD (+174%/+28%) for the phenotyping of crosses and 6.65/7.78 gSD (+95%/+82%) for the phenotyping of additional DH lines for the cross/DH traits, respectively. The genetic diversity in all 3 scenarios increased substantially to remain at around 60% of the genetic diversity of the initial breeding material with around 12% of all genotypes being heterozygous variants and only 35% of all markers being fixed after 30 cycles. The interested reader is referred to Supplementary Tables S1 and S2 for a detailed overview on the proportion of heterozygous variants and fixed markers for all scenarios and cycles.

Across replicates of the same scenario, standard deviations of simulation outcomes ranged between 1.30 gSD (baseline) and 1.69 gSD (phenotyping of crosses and added diversity), indicating high variance between replicates. However, as each scenario was simulated 250 times, the averages given here have a standard error of ¡ 0.1 for all scenarios, making all scenarios (except baseline vs. baseline with added diversity) statistically significantly different from each other (2-sample *t*-test; Student 1908), further highlighting the power of simulations to identify differences compared with a real-world setting with limited opportunity for replication. Note that significance in this context does not necessarily mean practical relevance.

**Fig. 4.**
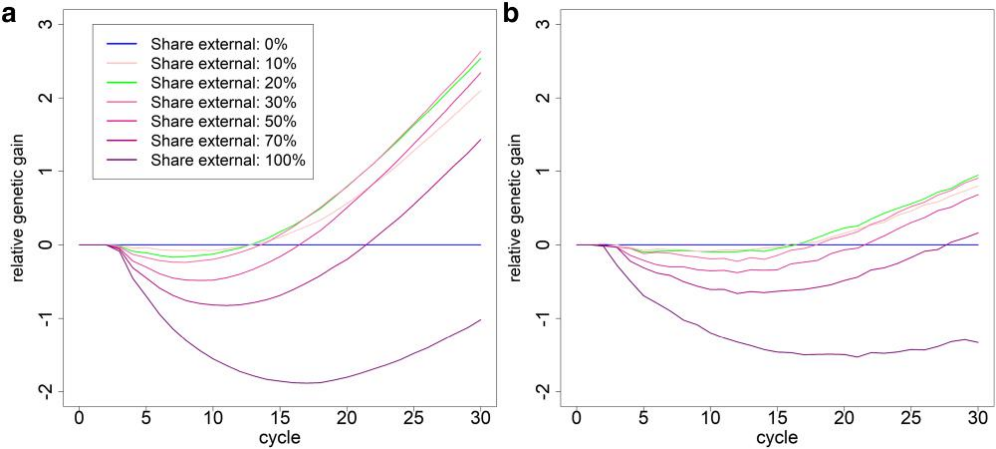
Genetic gain (in units of gSD) compared with the scenario with no newly introduced genetic material for the crosses a) and DH lines b) in the F2 lines of the respective cycle based on the used amount of genetic diversity from outside of the breeding program in each cycle.

When varying the share of introduced material in each cycle, the highest gains were achieved when 20 to 30% of the generated F2s included material from outside the breeding pool. Short-term gains were minimally higher with a lower share of newly introduced genetic material, while subsequent genetic gains dropped rapidly after 15 cycles (Fig. 4). A higher share of newly introduced material resulted in a lower short-term gain and similar per-cycle gains in later cycles. As expected, the levels of genetic diversity increased with a higher share of newly introduced genetic material (Supplementary Fig. S7).

### Genetic diversity through selection

All results on the impact of different selection intensities and mating allocation from AlphaMate are displayed relative to 3 basic scenarios: The baseline, additional phenotyping of crosses, and additional phenotyping of crosses with newly introduced genetic material.

#### AlphaMate

**Fig. 5.**
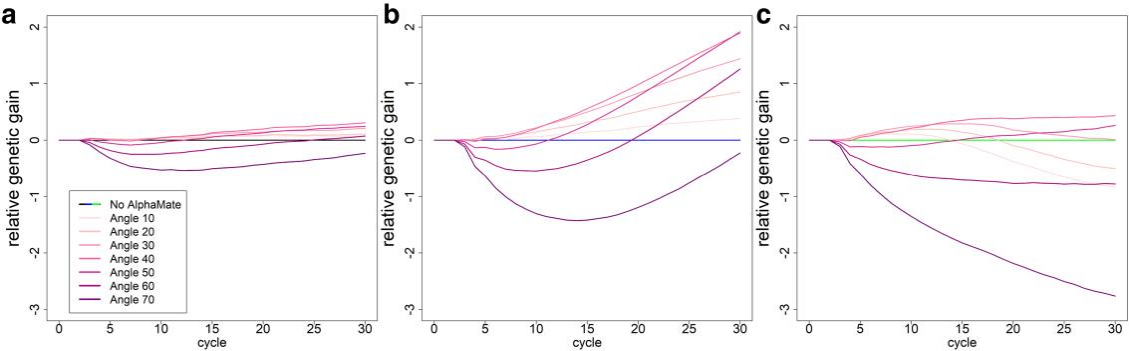
Genetic gain (in units of gSD) relative to the respective basic scenario based on the used angle between genetic gain and inbreeding in AlphaMate for the baseline scenario a), a scenario with phenotyping of the DH lines b), and phenotyped and introduction of genetic material in each cycle of the rapid cycle breeding scheme c). Coloring for reference scenarios is in concordance with Fig. 3.

For the scenarios without any introduction of new genetic material, the use of low angles in AlphaMate (Gorjanc and Hickey 2018) resulted in strict improvement compared with the respective basic scenario with both higher genetic gain (Fig. 5) and greater remaining genetic diversity (Supplementary Fig. S8). Higher angles resulted in substantially higher genetic diversity, however, even after 30 cycles this did not translate into a higher genetic gain for an angle of 70. The optimal angle for the baseline scenario was 40 with a genetic gain of 3.70 gSD (+9%) and 50 for the phenotyping of crosses (8.71 gSD; +29%). When introducing additional genetic material, relative genetic gains were low, the best performance in regard to genetic gain after 30 breeding cycles was observed with an angle of 40 leading to 9.77 gSD (+4%).

#### Selection intensity

**Fig. 6.**
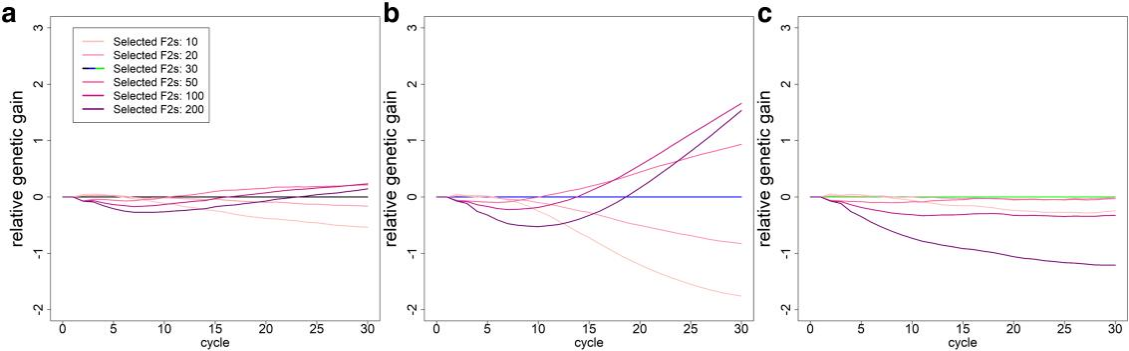
Genetic gain (in units of gSD) relative to the respective basic scenario depending on the selected F2 lines per cycle in the baseline scenario a), additional phenotyping of crosses b), and additional phenotyping of crosses with newly introduced genetic material c).

When selecting fewer F2 lines per cycle, we observed reduced short-term gains compared with the respective basic scenario (Fig. 6). However, after 5–7 cycles, the respective basic scenario already outperformed scenarios with 10/20 selected F2 lines with substantial drops in genetic gain (2.86/5.04/9.09 gSD; −15/−26/−3%). Contrary, a lower selection intensity scenario outperforms the basic scenario after 10–15 cycles when no additional genetic material was introduced with final genetic gains of 3.63 gSD (+7%) with no phenotyping and 8.45 gSD (+24%) with additional phenotyping of crosses after 30 cycles when selecting 100 F2s. The scenarios with newly introduced genetic material with selecting the top 20 or 50 F2 performed similarly to the basic scenario, while a substantially lower or higher number of F2 lines resulted in lower genetic gain (Fig. 6).

**Fig. 7.**
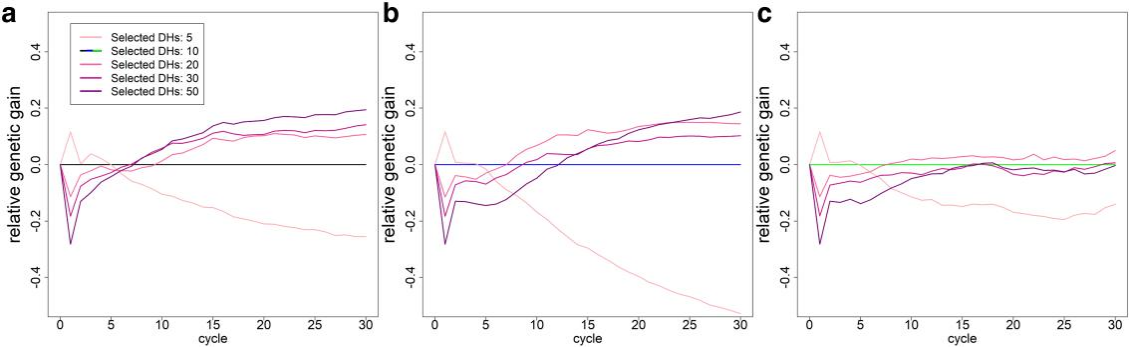
Genetic gain (in units of gSD) relative to the respective basic scenario depending on the number of initially selected DH lines to generate the C1F1 in the baseline scenario a), additional phenotyping of crosses b), and additional phenotyping of crosses with newly introduced genetic material c).

When only 5 DH lines were used to generate the initial C1F1 lines, we observed substantially higher gains in the first breeding cycle (+0.1 gSD in all 3 basic scenarios; Fig. 7). However, after just 2 breeding cycles, genetic gains were back on the same level as in the baseline scenario with 10 initially used DH lines, with substantially worse long-term performance for the 2 scenarios with no additionally introduced genetic material with gains of 3.15 / 6.29 gSD (−7 / −7 %). When adding no additional genetic diversity, the use of 50 founder DH lines resulted in long-term gains of 3.59/6.98 gSD, respectively (+6 / +3%). When introducing genetic diversity from outside of the breeding pool, founding population sizes of 10, 20, 30, and 50 DH lines to generate the initial C1F1 led to very similar genetic gains.

### Computing/performance specs

The baseline scenario required 250 s of computing time of which 174 s were spent on the generation of new individuals and 72 s for breeding value estimation. Additional phenotyping increased computing times for the breeding value estimation to 192 s. Adding additional genetic diversity took an additional 12 min. All computations were performed using a single core on a system with a 2X Xeon Platinum 9242 3.80 GHz processor. Peak memory usage was between 2.5 and 3.3 GB for all scenarios. Scenarios that used AlphaMate took 2.0 h of runtime with a singlerun of AlphaMate requiring about 4.0 minutes.

## Discussion

This study shows via stochastic simulations that rapid cycle breeding as suggested by Polzer *et al.* (2025) has the potential to be a valuable strategy in plant breeding to achieve substantial genetic gains in a limited time frame. However, to fully utilize its long-term potential, proper breeding program design and management of genetic diversity to ensure high prediction accuracies are of central importance. In the considered scenarios, prediction accuracies decrease after only a few breeding cycles and thus lead to significantly lower genetic gains. Thus, the generation of additional phenotypic data and the updating of the training model are of major importance. As correlations between DH and cross traits were high (0.6–0.8), phenotyping of either led to substantial improvements, with phenotyping of the crosses leading to higher gains for traits expressed as crosses and vice versa.

Depending on the breeding objective and time frame, the introduction of additional genetic diversity is of high importance to ensure long-term genetic progress (Hölker *et al.* 2019; Swarup *et al.* 2021). In case a rapid cycle scheme is “only” used for pre-breeding and to get new potential breeding material on an adequate level for introduction into an existing breeding scheme, this might not be necessary. For the here-considered maize rapid cycling scheme with genotypes from a European maize landrace, we observed higher or at least very similar genetic gains for the first 15 cycles of the scheme, without the introduction of new genetic material. This will of course be highly dependent on the species, initial genetic diversity, and selection intensity. Note that simulations tend to overestimate the reduction in genetic diversity (Pook *et al.* 2025) as trait definition and architecture are simulated to stay constant over time and mutations are only simulated to occur on included SNPs, while in reality diversity tends to reduce slower (Gerke *et al.* 2015). In principle, all this could be included in simulations, but to simplify simulations this was not considered in this study.

With regard to the used selection intensity, resulting from the number of initial used DH lines and the number of selected F2 lines per cycle, our study suggests that the efficiency of this strategy depends on whether additional genetic diversity was added to the breeding pool at later time points. When applying weaker selection, genetic gains were slightly lower for 5–10 cycles when no additional genetic material is added to the breeding pool (e.g. pre-breeding), while substantial long-term improvements were obtained. In breeding schemes for variety development, where new material is frequently added, the negative long-term effects of high selection pressure are much lower. Note here that factors like lethal mutations or passive selection due to lines not being able to produce DH lines or inbreeding depression were not considered in the simulations (Holland 2007; Sitonik *et al.* 2019; Zeitler *et al.* 2020).

In particular, when no additional genetic diversity was added to the breeding program, the use of optimum contribution selection via AlphaMate (Gorjanc and Hickey 2018) was highly beneficial as it led to higher genetic gains and more remaining genetic diversity. Thus, it represents a strict improvement of the breeding scheme without any disadvantages (besides minor computing costs). When using AlphaMate with an angle of about 40, we found the best overall performance. Slightly lower angles produced a larger short-term gain and slightly higher angles produced a larger long-term gain. Effects were smaller in the scenario in which new material was introduced in the breeding scheme, which might be caused by limited links between relationships (which would even be more severe when using pedigree-based relationships) and the higher overall remaining diversity even without the use of optimum contribution selection. Other approaches for mate allocation were not tested as they did not provide the required flexibility of equal contributions of all parents to the next cycle (Meuwissen 1997), are not really designed for plant breeding applications with single-sex and their mating schemes (Wellmann 2017) or should result in very similar mating plans as they are methodologically similar (Kinghorn 2011).

The traits design used in this study with 3 PH traits is a simplification of reality, as plant height PH will usually not be the primary target trait. However, the results presented here should generalize to more sophisticated indices with more traits or non-linear effects. Here, all traits were considered polygenic, with predominantly additive and dominant QTLs and only a small amount of epistatic variation. Note here that trait architectures are highly trait dependent, with our choice being a more or less arbitrary choice in line with estimates from Rogers *et al.* (2021) with a broader range of estimates for various traits provided by Monir and Zhu (2018). This choice was also made to include various QTL types while not requiring a specific breeding strategy (Holland 2001; Ali *et al.* 2020) or prediction model (Martini *et al.* 2017; Vojgani *et al.* 2021) for epistasis, keeping simulations simple.

We would assume that scenarios with different selection intensities or selection criteria should be impacted by such changes in trait architecture in a very similar way, thus not compromising the overall conclusions of this study. Note that differences in trait architecture might result in different scenarios being affected in different ways when phenotyping strategies or other major breeding program design changes are considered, e.g. with lower correlations between DH and cross traits, higher share of dominance or epistasis (Khattab *et al.* 2010; Zaazaa *et al.* 2012), individual QTL explaining a high share of variation (Salvi *et al.* 2007) or multiple environments (de Leon *et al.* 2016). Therefore, the results of the simulation study need to be taken with caution for more complex breeding schemes. Nonetheless, it should easily be possible to adapt simulations to be more in line with reality in the given context.

This study represents the first published applications of MoBPS XREFX on a concrete plant breeding scheme (Pook *et al.* 2020b). While the outcomes may align with theoretical expectations, their quantification confirms the robustness of the simulation framework. Note that the added value of stochastic simulations does not solely lie in the result that a high selection intensity will lead to higher short-term genetic gains and lower long-term gains as the genetic diversity is strongly reduced early. Instead, stochastic simulations should be seen as a way to quantify the size of such effects without requiring assumptions on factors like the accuracy of the breeding value estimation, the remaining genetic diversity, or the effect of using a certain selection technique, as those will be implicitly calculated in the process of simulations (Hassanpour *et al.* 2023). Thus, providing breeders with a powerful tool to identify inefficiencies and optimize breeding program design. Although general observations of the results reported here should translate well to other breeding schemes and/or crops, the exact optima will depend on the population structure, trait architectures, and the design of the breeding scheme.

## Data Availability

Genotype data for the founder DH lines were taken from Mayer *et al.* (2020) with the data being processed as described in Pook (2019). The data are publicly available at https://doi.org/10.6084/m9.figshare.12137142. Although the dataset contains 501,124 useable markers, this was down-sampled to reduce the number of markers to 50,000 and mimic a medium-density array. The source code for all simulations and the MoBPS R package can be found at https://github.com/tpook92/MoBPS. MoBPS version 1.9.06 was used for all simulations in this study. The software AlphaMate is available at https://github.com/AlphaGenes/alphamate. AlphaMate version 0.2.0 was used for all simulations performed. Supplementary files are available at FigShare: https://doi.org/10.25387/g3.28804310 Supplementary Fig. S1 provides a schematic overview of the rapid cycle breeding scheme with blue boxes indicating cohorts of lines with numbers indicating the number of lines included in a particular cohort. This visualization was generated via the MoBPS interactive environment from Pook *et al.* (2021a), Supplementary Fig. S2 with additional phenotyping of F2 lines, Supplementary Fig. S3 with additional phenotyping of DH lines and Supplementary Fig. S4 with newly introduced additional genetic material. Supplementary Fig. S5 provides information on the accuracy of the breeding value estimation for the F2 lines in the different breeding cycles for each of the 3 traits in the baseline scenario. Supplementary Fig. S6 provides an overview of prediction accuracies per trait and breeding cycle for the different scenarios. Supplementary Fig. S7 provides a comparison of the remaining genetic diversity for the different base scenarios depending on the amount of genetic material from outside of the breeding program introduced in each cycle. Supplementary Fig. S8 provides a comparison of the remaining genetic diversity for the base scenarios depending on the angle used in AlphaMate (Gorjanc and Hickey 2018) in each cycle. Supplementary Table S1 provides information on the share of heterozygous variants for all scenarios and cycles. Supplementary Table S2 provides information on the share of fixed markers for all scenarios and breeding cycles.
